# Understanding Aβ Peptide Binding to Lipid Membranes: A Biophysical Perspective †

**DOI:** 10.3390/ijms25126401

**Published:** 2024-06-10

**Authors:** Hasna Ahyayauch, Massimo E. Masserini, Alicia Alonso, Félix M. Goñi

**Affiliations:** 1 Departamento de Bioquímica, Instituto Biofisika (CSIC, UPV/EHU), Universidad del País Vasco, 48940 Leioa, Spain; ahyayauch@hotmail.com (H.A.); alicia.alonso@ehu.eus (A.A.); 2 Institut Supérieur des Professions Infirmières et Techniques de Santé, Rabat 60000, Morocco; 3 Laboratoire de Biologie et Santé, Unité Neurosciences, Neuroimmunologie et Comportement, Faculty of Sciences, Ibn Tofail University, Kénitra 14000, Morocco; 4 School of Medicine and Surgery, University of Milano-Bicocca, 20900 Monza, Italy; massimo.masserini@unimib.it

**Keywords:** Aβ42, β-amyloid, Aβ membrane binding, ganglioside, sphingomyelin, cholesterol, isothermal calorimetry, Langmuir balance, Alzheimer’s disease

## Abstract

Aβ peptides are known to bind neural plasma membranes in a process leading to the deposit of Aβ-enriched plaques. These extracellular structures are characteristic of Alzheimer’s disease, the major cause of late-age dementia. The mechanisms of Aβ plaque formation and deposition are far from being understood. A vast number of studies in the literature describe the efforts to analyze those mechanisms using a variety of tools. The present review focuses on biophysical studies mostly carried out with model membranes or with computational tools. This review starts by describing basic physical aspects of lipid phases and commonly used model membranes (monolayers and bilayers). This is followed by a discussion of the biophysical techniques applied to these systems, mainly but not exclusively Langmuir monolayers, isothermal calorimetry, density-gradient ultracentrifugation, and molecular dynamics. The Methodological Section is followed by the core of the review, which includes a summary of important results obtained with each technique. The last section is devoted to an overall reflection and an effort to understand Aβ-bilayer binding. Concepts such as Aβ peptide membrane binding, adsorption, and insertion are defined and differentiated. The roles of membrane lipid order, nanodomain formation, and electrostatic forces in Aβ–membrane interaction are separately identified and discussed.

## 1. Introduction

The neurodegenerative disorder known as Alzheimer’s disease (AD) is the most frequent cause of late-age dementia [1]. It is characterized by a progressive loss of memory and cognitive abilities. Plaques were detected on central nervous system structures from the very early descriptions of the disease, but it was only in 1984 when Glenner and Wong [2] put forward the idea that the cause of AD could be the accumulation of the amyloidogenic peptide Aβ. These authors showed that this serum peptide, which they had isolated, would be the chief component of amyloid plaques. An amyloid precursor protein (APP) is sequentially processed by β- and γ-secretases [2,3], giving rise to the pathogenic Aβ peptide. Aβ contains 39–43 amino acid residues. The most common isoforms are Aβ40 and Aβ42, and the latter is considered to be particularly pathogenic [4]. Excessive APP cleavage or insufficient Aβ clearance would give rise to the accumulation of self-aggregating Aβ peptides. The amyloid hypothesis is now the dominant model of AD pathogenesis, and it has given rise to an impressive amount of research work (see Tempra et al. [3] for a review).

The formation of plaques by the Aβ peptide involves the transformation from its monomeric form to an aggregated fibrillar species. Initially, the Aβ peptide is secreted from cells in a soluble state, but it gradually aggregates, forming oligomers, multimers, and fibrils, eventually resulting in extracellular plaque deposits. Numerous studies suggest that soluble Aβ aggregates, particularly oligomers and protofibrils, play a crucial role in Alzheimer’s disease (AD) dementia by causing synaptic dysfunction, neuronal stress, the propagation of tau pathology, and cell death. These oligomers and protofibrils can further aggregate into insoluble β-pleated sheets, forming mature amyloid fibrils and plaques. It has been proposed that the formation of these insoluble fibrils and plaques might serve as a protective mechanism to reduce the toxicity of oligomers. Research has shown that cell membranes significantly accelerate Aβ aggregation (see, for example, [4,5,6,7] for reviews). Many of these studies, including those conducted by our laboratories [8,9,10,11,12], have investigated the interaction of β-amyloid with model membranes. The general consensus is that Aβ interacts with lipid surfaces, especially those containing anionic lipids, leading to a much faster aggregation rate compared to its behavior in a solution.

**Figure 1:**
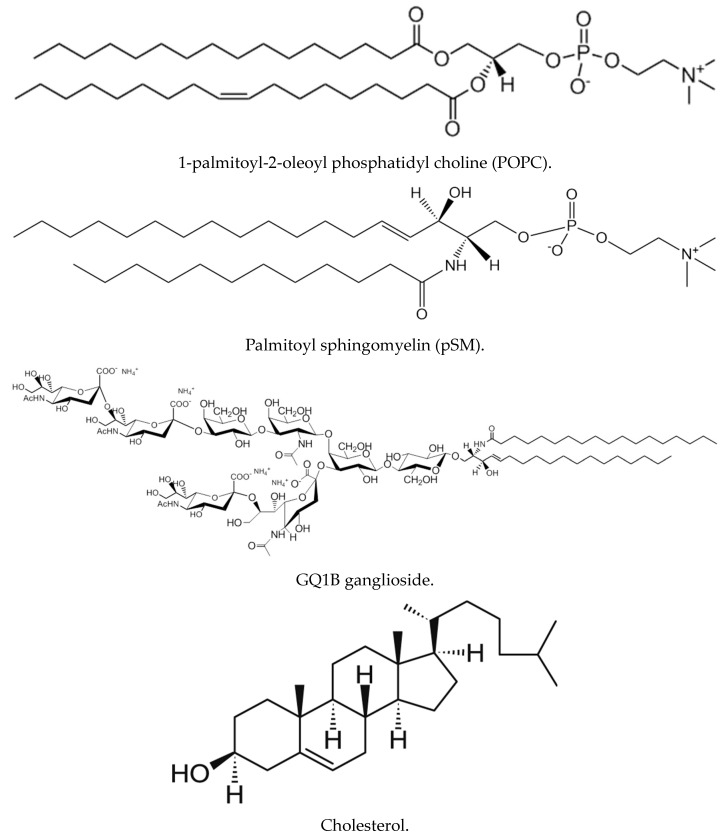
The structures of the main lipids involved in the studies discussed in the present review.

The present study aims to review the most relevant studies on the interaction of the Aβ peptide with lipids in model systems, monolayers, or bilayers. Relevant lipid structures are shown in Figure 1. Some open questions to be dealt with are, e.g., the conceptual and experimental distinction between peptide binding to adsorption onto and insertion into membranes, the surface catalysis of peptide aggregation, the influence of bilayer physical properties (physical state or phase, fluidity, molecular order, or electrostatic charge) on peptide–lipid interaction, the suitability of the experimental methods used, and the pathophysiological relevance of the biophysical studies with model membranes. For simplicity, in this review, “binding” refers to any form of detectable peptide–lipid interaction unless otherwise indicated. Aβ, Aβ peptide, and amyloidogenic peptide Aβ will be used as synonyms.

## 2. Lipid Phases and Their Significance

To fully understand the interaction between Aβ peptides and lipids, it is essential to discuss the concept of lipid phases. A phase is defined as a region where all physical properties of a material are uniform. In the past century, several phases with properties intermediate between liquid and solid were identified; they are collectively known as mesophases. A common example is the liquid crystalline phase in which cell membranes mostly exist. This phase exhibits liquid-like fluidity while maintaining a crystal-like molecular orientation. Lipids dispersed in water can form various mesophases, and they are said to be mesomorphic [13].

The best methods for describing lipid phases in aqueous environments include X-ray scattering [14,15,16], 31P NMR [17], and differential scanning calorimetry [18,19]. The main mesophases adopted by pure membrane lipids in water are the lamellar (L), micellar (M), inverted hexagonal (H_II_), and inverted cubic (Q_II_) phases [20]. The lamellar (L) phases, consisting of two lipid layers with their non-polar regions in contact and away from water, are spontaneously adopted by most phospholipids and glycolipids. Saturated membrane lipids can form a gel (solid) L_β_ lamellar phase at a certain temperature and a fluid (liquid-crystalline) L_α_ phase at a higher temperature. There is also a liquid-ordered (L_o_) lamellar phase [21], which is formed in the presence of saturated phospholipids and cholesterol, where lipid molecules have lateral diffusion but restricted rotation around alkyl chain C-C bonds.

## 3. Model Membranes: Monolayers and Bilayers

To clarify some methodological and conceptual aspects that are often unclear in the literature on Aβ peptide–lipid interactions, we discuss lipid monolayers and bilayers. Studying these, either in situ or after transferring them to solid supports, is crucial for understanding lipid behavior and lipid–protein interactions in membranes.

### 3.1. Monolayers

Lipid monolayers, spread over an air–water interface, can model various physical phenomena that are seen, or presumed to occur, in biomembranes [8]. These include phase transitions, changes in lateral diffusion, alterations in lateral compressibility/elasticity, and interactions that can influence lipid mixing, resulting in critical points as well as in immiscible or coexisting lateral phases (domains) [12,22,23,24,25]. Early studies focused on constructing lipid phase diagrams with mixtures of two lipid components [23,24,26], but more recent research has examined the phase behavior of “raft” ternary mixtures of phosphatidyl choline/sphingomyelin/cholesterol (PC/SM/Chol) using epifluorescence microscopy to monitor lipid distribution patterns [27,28,29,30].

Monolayers can be transferred from the air–water interface to solid supports, allowing them to be studied using techniques such as epifluorescence microscopy [31,32], atomic force microscopy [33,34,35], and imaging mass spectrometry [36,37,38]. Ternary mixtures of PC/SM/Chol have been analyzed in detail, revealing a domain formation enriched in sphingomyelin and cholesterol, and underlining the importance of acyl composition [36,37,38].

### 3.2. Bilayers

Lipids can also form bilayers, with their hydrophobic and hydrophilic moieties being organized into double layers. This occurs with phospholipids, glycolipids, and sterols. In aqueous media, lipids arrange themselves with hydrophobic portions facing inward and polar moieties facing outward, forming bilayers spontaneously, such as during liposome formation. This bilayer arrangement thermodynamically stabilizes the molecules, which are neither entirely hydrophobic nor hydrophilic [20,39].

Membrane proteins associate with the lipid bilayer either as peripheral proteins attached to the polar headgroups or as integral proteins embedded in the hydrophobic matrix. Peripheral proteins [40] can be detached using mild methods like changes in the buffer pH or ionic strength, whereas integral proteins require harsher treatments like detergents or organic solvents.

## 4. Biophysical Methods in the Study of Aβ Peptide Interaction with Membranes

A range of experimental and numerical methods are available for studying Aβ–lipid interactions. A selection of the most widely used ones is presented below.

### 4.1. The Langmuir Balance and Langmuir Monolayers

**Figure 2:**
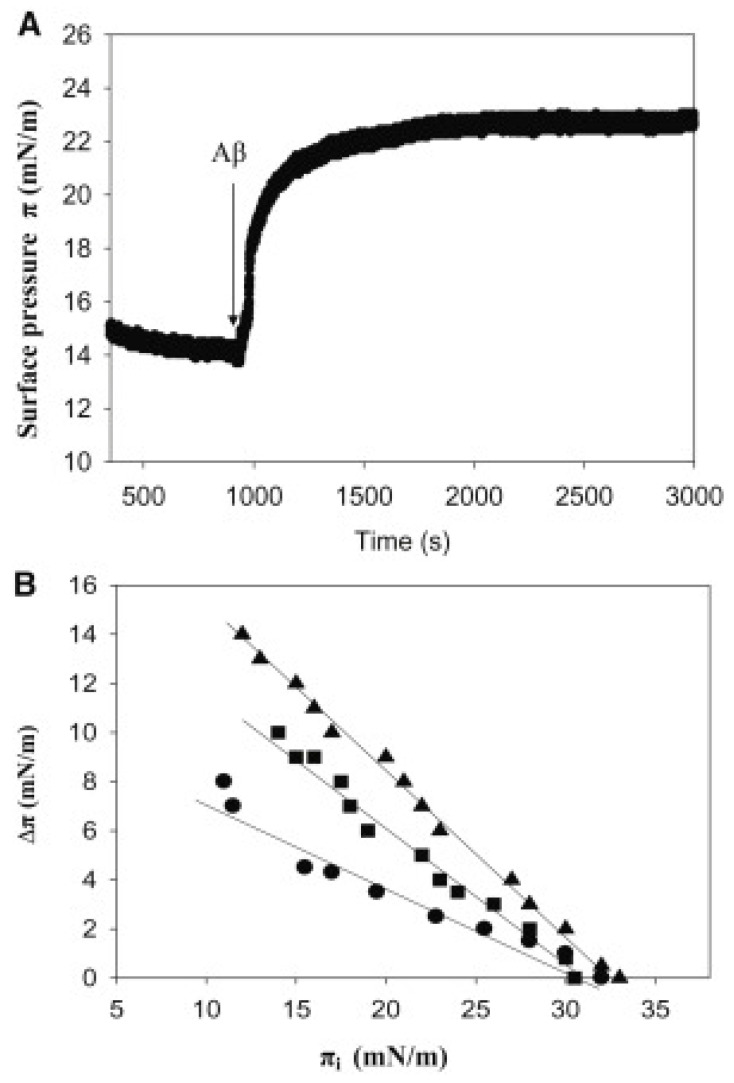
Changes in surface pressure of lipid monolayers upon insertion of Aβ42 monomers at varying initial pressures. (**A**) Representative experiment obtained with SM/Chol/DMPA (40/40/20) at 15 mN/m. (**B**) Equilibrium values. (●) SM/Chol (1:1); (▲) SM/Chol/DMPA (47.5/47.5/5); (■) SM/Chol/DMPA (40/40/20). Average values ± SE (*n* = 3). Aβ42 stock solution was 50 mM. Aβ42 final concentration in the trough was 1.22 mM. (Reprinted with permission from [8], 2012, Elsevier).

Surface pressure experiments are performed using a Langmuir trough with continuous stirring. The aqueous phase is composed of a buffered saline solution. The chosen lipid or lipid mixture, dissolved in a chloroform/methanol solution (2:1), is gently applied to the surface until the target initial surface pressure is reached. Aβ binding to lipid monolayers is assessed at an initial surface pressure π_i_ that exceeds the maximum Δπ induced by the adsorption of pure Aβ at an air–water interface [8]. The protein is introduced into the subphase using a micropipette through a designated opening. The increase in surface pressure over time is recorded until a stable reading is achieved (Figure 2).

Once a Langmuir monolayer is established at the air–water interface, various reflection techniques can be employed. Grazing incidence X-ray diffraction provides insights into peptide-induced alterations in the lipid monolayer [41,42]. Infrared reflection absorption spectroscopy (IRRAS) enables the measurement of the conformation and orientation of the peptide adsorbed at the air–water interface and within the lipid monolayer [41,42]. Brewster angle microscopy is used to detect fibrils within the monolayers [43,44]. Additionally, when Langmuir monolayers are transferred onto solid inert surfaces like mica, atomic force microscopy can be utilized to uncover new details of Aβ–lipid interactions [43,44]. Finally, fluorescence techniques are combined with monolayer experiments to further analyze these interactions, as discussed in Section 4.6.

### 4.2. Sucrose Gradient Ultracentrifugation

Liposome flotation assays are a direct method for measuring the binding of proteins to lipid vesicle membranes. Vesicles that have proteins bound to them are denser and sediment more readily, while those without proteins tend to float to the top of the gradient. This technique is particularly useful because it allows liposomes to mimic the composition and curvature of cell membranes as they occur in vivo. Initially, Masserini and colleagues used ultracentrifugation to study the interaction of anti-Aβ monoclonal antibody-decorated nanoliposomes with Aβ peptides [45].

More recently, Ahyayauch et al. extensively employed this technique to examine the binding of Aβ42 to liposomes containing gangliosides [9]. In their studies, a fixed concentration of pure Aβ (typically around 15 μM) was incubated with liposomes at a 1:200 protein-to-lipid molar ratio. The mixture was then subjected to equilibrium sucrose gradient centrifugation, which allows protein-free vesicles to float. This enables the quantification of the protein bound to the liposomes. To facilitate detection, rhodamine-stained liposomes (0.5 mol % Rho-PE) are commonly used.

### 4.3. Calorimetric Methods

**Figure 3:**
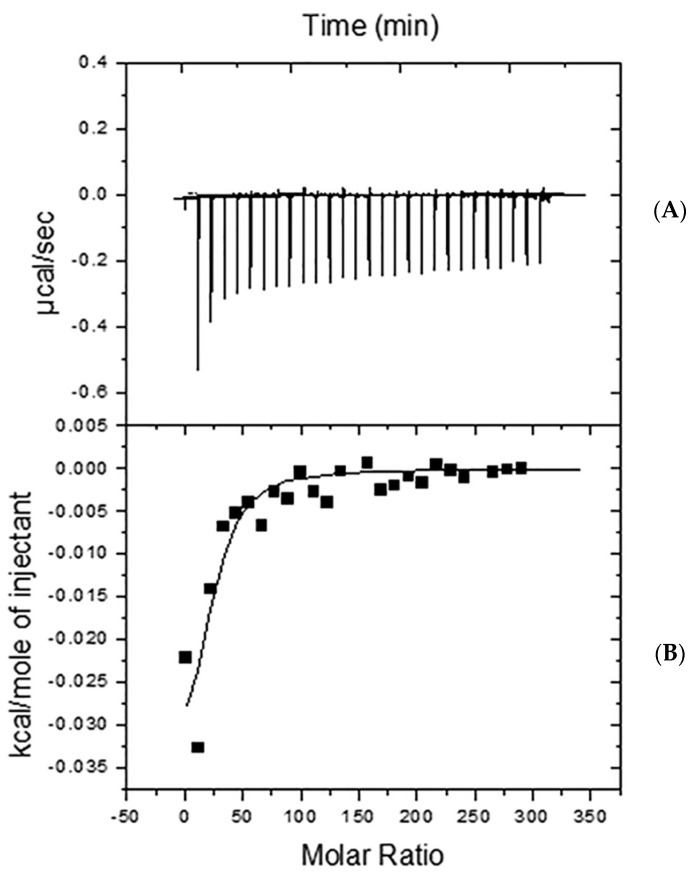
ITC calorimetric studies. (**A**) A representative titration calorimetry curve of unilamellar vesicles composed of SM/Chol/GT1b ganglioside (47.5/47.5/5 mol ratio) with Aβ42 peptide fibrils. The calorimetric trace was observed upon successive injections of lipid vesicles into an Aβ42 solution contained in the reaction cell. (**B**) Cumulative heats of reaction obtained from the integration of the peaks are displayed in the top plot. The solid line represents the fitting of the experimental data to a partitioning model. The calorimetric cell was filled with a 28 μM Aβ42 solution. Lipid vesicles at a 35 mM lipid concentration were injected into the cell (1.43 mL) in 10-μL steps, leading to a 143-fold dilution of lipid vesicles. The titration experiments were performed at 37 °C. (Reprinted with permission from [11], 2021, Elsevier).

The primary calorimetric technique employed in investigating Aβ–lipid interactions is isothermal calorimetry (ITC) (Figure 3). Specifically, researchers often use high-sensitivity ITC to measure the enthalpy change associated with the partitioning of Aβ42 into lipid vesicles, typically large unilamellar vesicles (LUVs). In this method, the calorimetric cell is filled with an Aβ solution, and lipid vesicles are injected into the cell. The injections are typically spaced at 10 min intervals and at a rate of 2 s/μL while maintaining constant stirring throughout the experiment. As each lipid injection occurs, free Aβ peptide monomers partition into the bilayer membrane, and the resulting heat of reaction is measured. The heat of reaction decreases progressively as less peptide remains free in the solution (Figure 3). By integrating each calorimetric peak, researchers determine the heat of reaction, which is then used to derive the thermodynamic parameters of partitioning [7,8,46,47,48]. A model of partition equilibrium of the peptide molecules between the aqueous phase and the membrane is assumed for this interaction [47].

Another calorimetric approach is differential scanning calorimetry (DSC), which is commonly used to detect thermotropic phase transitions in lipid–water systems and protein thermal denaturation. Martinez-Senac et al. [49] utilized DSC to illustrate that the impact of Aβ25–35 on the gel-to-fluid transition was minimal at a neutral pH for negatively charged phospholipids and practically non-existent for DMPC. Additionally, these authors inferred, based on their findings, that peptide–lipid interactions were predominantly electrostatic in nature and favored peptide aggregation. DSC was also employed, without peptides, to investigate the phase behavior of sphingomyelin vesicles containing GM1 ganglioside and cholesterol [50].

### 4.4. Thioflavin T (ThT) Fluorescence Assays

Thioflavin T (ThT) fluorescence is a straightforward and frequently utilized technique, chosen more for its simplicity than for its analytical potential. ThT fluorescence increases in proportion to the β-sheet content of peptides [51,52], making it suitable for a semi-quantitative analysis of monomer–oligomer transitions or, more generally, monomer–aggregate transitions of Aβ peptides in solvents. These transitions often involve a shift from helical (or random) to β-sheet conformation. To conduct ThT fluorescence assays, ThT is dissolved in glycine (50 mM, pH 8.2), filtered (0.22 µm), and added to incubation mixtures typically containing 5 μM Aβ and a 1:200 peptide-to-lipid mole ratio at 37 °C. Fluorescence is then measured using a spectrofluorometer (λ_ex_ = 446 nm, λ_em_ = 485 nm). Control samples consisting of pure Aβ and peptide-free vesicles are also prepared and measured accordingly. Numerous studies have employed ThT fluorescence to investigate β-sheet formation in Aβ peptides [8,9,10,11,43,44,53].

### 4.5. Computational Methods

Computational methods, exemplified by the methodology described by Ahyayauch et al. [8,10], play a significant role in these studies. Starting coordinates for the protein structure are obtained from databases such as PDBid 1IYT for Aβ42 [54], which provides a three-dimensional structure obtained via NMR, revealing two helical regions (residues 8–25 and 28–38) connected by a type I β-turn. Aβ42 is modeled with ionized C- and N-termini in a zwitterionic structure. The initial protomer from the PDB entry is positioned approximately 1 nm from the surface of a bilayer composed of 128 lipids, with coordinates obtained from previous sources [55,56]. The simulation systems are immersed in simulation boxes containing SPC water and 150 mM NaCl, and simulations are conducted using the GROMACS 4.6.7 package [57]. Energy minimization and equilibration steps are followed by molecular dynamics production runs using the GROMOS53a64 force field [58]. For lipid descriptions, a combination of force field parameters is used, with specific parameters for sphingomyelin, other phospholipids, and cholesterol [59,60,61]. Simulations are performed at 298 K, and a dual resolution solvent approach is implemented as described in the literature [62,63]. Molecules beyond 1 nm of the protein or membrane are substituted by coarse-grained (CG) water [64]. All simulations are performed with a time step of 2 fs [65] with a direct cut-off of 1 nm. Temperature coupling is achieved with the V-rescale algorithm [66]. Pressure coupling to 1 atm is obtained using the Parrinello-Rahman algorithm [67]. Trajectories are analyzed using visualization software such as VMD, version 1.9.4 [68].

### 4.6. Miscellaneous Techniques

The methods discussed earlier, particularly Langmuir monolayers, isothermal calorimetry, and molecular dynamics, have been extensively employed and have provided detailed insights into the interactions between Aβ peptides and lipid molecules in membranes. However, several other techniques, although less informative or less commonly used, warrant discussion.

*Circular dichroism (CD)* relies on the differential absorption of left- and right-handed polarized light and serves as an indicator of the optical activity of different secondary structure elements in a protein. While CD is primarily utilized to detect conformational changes in peptides/proteins in solution, its quantitative information is limited to estimating the percentage of amino acid residues involved in α-helix conformation. Although CD is more commonly used in solution studies of Aβ, it has provided valuable insights into Aβ oligomerization [7,69,70,71,72] and can complement ThT fluorescence measurements.

*Nuclear magnetic resonance (NMR)* has been sporadically applied to study Aβ peptide–lipid interactions. This technique involves orienting the spin of a nucleus (e.g., 1H, 2H, or 31P) in an external magnetic field and provides information on nuclear mobility and intra- and inter-molecular motions. While NMR requires minimal chemical modification of lipids, its sensitivity is relatively low. Some studies have utilized solid-state NMR to investigate the lipid molecular structure in the presence of Aβ peptides [69,73,74].

*Electron spin resonance (ESR)*, akin to NMR but involving the spin of an electron, requires a chemical modification of lipids with spin probes, such as TEMPO. ESR has been utilized to understand how ω-3 fatty acids regulate the interaction of Aβ peptides with lipid membranes [75].

*Fluorescence techniques*. Fluorescence methods exploit the phenomenon wherein an atom or molecule absorbs the radiation of a specific wavelength and subsequently emits radiation of a longer wavelength (lower energy) after a brief delay of nanoseconds. This property of fluorescence has numerous applications in the field of biology. In our research context, Chi et al. [76] utilized Texas Red 1, 2-dihexadecanoyl 3-phosphoethanolamine (TR-DHPE) to selectively label specific domains within monolayers. They observed dark patches representing condensed domains, where bulky head-group-labeled TR-DHPE dye molecules were excluded, alongside bright regions representing the liquid expanded phase, where the dye predominantly localized. At lower GM1 concentrations, Aβ displayed a preference for inserting into the disordered, liquid expanded phase. Conversely, at higher GM1 concentrations, Aβ insertion occurred more uniformly across the monolayer, leading to no discernible preferences for either the expanded or condensed phase. Lin et al. [77] employed dynamic (time-resolved) fluorescence imaging of monolayers to elucidate the interaction between Aβ40 and lipid monolayers containing negatively charged lipids and cholesterol. Additionally, Hossain et al. [53] investigated the intrinsic fluorescence of tyrosine residues in the Aβ42 peptide and explored the effects of docosahexaenoic acid on amyloidogenesis. They observed that this polyunsaturated ω-3 fatty acid reduced the levels of tyrosine intrinsic fluorescence, implying decreased fluidity in the microenvironment and a certain degree of protection against amyloidogenesis.

## 5. A Review of the Selected Results

### 5.1. Monolayer Studies

The earliest investigations into the interaction between Aβ peptides and lipid monolayers were probably conducted by Seelig and colleagues in 1997 [69]. They observed that Aβ40 could effectively penetrate acidic monolayers when the lateral pressure was low (20 mN/m). The degree of integration notably increased with the acidic lipid content in the monolayer. However, no insertion of Aβ40 was detected at a lipid packing density equivalent to that of a bilayer (lateral pressure ≥32 mN/m). These findings were subsequently confirmed and expanded upon by Maltseva et al., who used monolayers consisting of phosphatidyl ethanolamine [41] or various zwitterionic or negatively charged phospholipids [42]. Thakur et al. [78] further explored the intricate effects of cholesterol, in combination with several phospholipids, on the binding of Aβ40.

Ahyayauch et al. [8] delved into the initial stages of Aβ42 deposition on membranes. Aβ42 is deemed more pathogenic than Aβ40. In the absence of lipids, the injection of Aβ42 into the aqueous phase induced an increase in surface pressure, which reached equilibrium after approximately 1 h. This indicated that Aβ42, like many other peptides, was surface active [79]. The rise in surface pressure was dose-dependent and reached a plateau at around 10 mN/m for Aβ concentrations slightly exceeding 1 μM. Consequently, at these concentrations and higher, the interface became saturated with adsorbed peptide, and the peptide partitioned between the interface and the bulk water [79]. Previous studies have reported plateau values ranging from 12 to 17 mN/m with Aβ42 or Aβ40 [62,63,64,66], although the source of this variability remains unclear.

To assay Aβ insertion into lipid monolayers, a separate series of experiments were conducted. In these experiments, a lipid monolayer was extended at the air–water interface, and the peptide was injected into the aqueous subphase. The initial surface pressure of the lipid monolayer was set as desired at values exceeding 10 mN/m to prevent simultaneous peptide insertion and adsorption. The insertion of Aβ into the lipid monolayer at the interface induced a further increase in surface pressure Δπ (see Figure 2A). As the initial pressure increased, Δπ decreased (see Figure 2B) until a threshold was reached, typically at 30–33 mN/m for this system, beyond which peptide insertion ceased. The inclusion of the negatively charged dimyristoyl phosphatidic acid (DMPA) in the monolayer lipid composition facilitated peptide insertion in the 10–30 mN/m range, with the order of ease being SM/Chol (1:1) < SM/Chol/DMPA (40:40:20) < SM/Chol/DMPA (47.5:47.5:5), all given as mole ratios. However, the maximum initial pressure for all three lipid compositions was approximately 30 mN/m, which was assumed to be the average surface pressure in cell membranes, albeit with considerable fluctuations. Terzi et al. [69] and Maltseva et al. [41,42] similarly found this maximum initial pressure of 30 mN/m for Aβ40. This suggests that, in a hypothetical cell membrane domain in the liquid-ordered state, Aβ would exist in equilibrium between the free and membrane-bound forms. The facilitation of insertion by the presence of negatively charged lipids in the monolayer supports the role of electrostatic interactions in stabilizing Aβ42 insertion into lipid monolayers, which is consistent with prior propositions [41,78]. A compression isotherm of the SM/Chol/DMPA (40/40/20) mixture indicated an average area per molecule of approximately 0.4 nm^2^, which is in agreement with MD calculations (see Section 5.3 below).

Ahyayauch et al. expanded the applications of Langmuir monolayers to investigate the lipid interactions of Aβ42 [9,10,11,12]. They examined the binding of A42 peptide monomers to sphingomyelin/cholesterol (1:1 mol ratio) monolayers containing 5 mol% gangliosides (GM1, GT1b, or a mixture of brain gangliosides) [10]. Generally, gangliosides facilitated monolayer–peptide binding, with peptide insertion becoming easier in the following order: SM/Chol < SM/Chol/total gangliosides ≈ SM/Chol/GT1b < SM/Chol/GM1 in the 10–30 mN/m range. However, the limiting initial pressure for all four lipid compositions was approximately 32–34 mN/m. These findings, independent of the geometric fluctuations produced by gangliosides in bilayers, confirmed that gangliosides facilitate the binding of Aβ42 to membrane lipids. In another series of experiments, four different lipid compositions were tested in monolayer form, namely POPC with 0, 1, 3, and 5 mol% GM1. Peptide binding became easier with an increasing ganglioside concentration in the 12–22 mN/m range, with the limiting initial pressure for all three lipid compositions being close to 22 mN/m. Considering that 30 mN/m is the supposed average surface pressure in fluid disordered bilayers, the data suggest that in such bilayers, Aβ42 would adsorb onto the lipid surface without being able to become inserted in the bilayer.

Ahyayauch et al. also examined the state of aggregation of Aβ42 (monomer, oligomer, or fibril) and its influence on the interaction with sphingomyelin/cholesterol (with or without gangliosides) in lipid monolayers [11,12]. They found that all three peptide preparations could be inserted into lipid monolayers of any composition and initial π in the 10–30 mN/m range, although fibrils were less capable of doing so than oligomers or monomers, with their maximum initial π value being ≈25 mN/m. Comparing various lipid compositions, monolayers lacking gangliosides were the most resistant to peptide insertion, while GM1 ganglioside allowed for easier insertion over a wide range of πi. The effect of ganglioside concentration in the monolayers was nuanced, with GM1 ganglioside concentrations below 5% not clearly favoring peptide insertion compared to ganglioside-free SM/Chol mixtures. Additionally, variations in the monolayer electric charge showed that Aβ42 aggregation hindered peptide insertion, with the lipid composition causing minimal differences in insertion, except for a slight facilitation of monomer and oligomer insertion by gangliosides. Notably, SM/Chol exhibited particularly low binding to fibrils.

Meanwhile, Fidelio and colleagues have also reported intriguing results on the interaction of Aβ40 fibrils with lipid monolayers [43,44,80,81]. Alvarez et al. examined the surface properties of Aβ40 amyloid peptides mixed with POPC or DSPC phospholipids in Langmuir monolayers [43]. They observed the formation of a fibril-like structure when mixed with POPC but not with DSPC at low amyloid peptide proportions. Subsequent studies demonstrated a “dynamical smelting” process when pre-formed fibrils were laterally mixed with gangliosides [44]. Alvarez et al. described how Aβ40 fibrils altered the topography and mechanical properties of lipid membranes, inducing a compressional hysteresis in the film. These authors further investigated the surface properties of DPPC and Aβ40 mixed monolayers at different temperatures, revealing that fibril-like structures of Aβ40 were triggered, specifically in the liquid-expanded region of DPPC films as they were compressed.

### 5.2. Isothermal Calorimetric Studies

The earliest calorimetric investigations of Alzheimer’s beta-amyloid fragments were conducted by Seelig and colleagues in 1994 [70]. Initially, they explored the random coil–β-sheet transition of a short fragment (residues 25–35) using ITC, estimating an enthalpy of association (ΔH) of approximately −3 kcal/mol. Subsequently, they studied the binding of Aβ peptides to lipid vesicles containing negatively charged lipids [71], observing a shift in the random coil–β-sheet equilibrium towards the β-sheet structure in the presence of lipids. The Aβ(25–35)OH peptide exhibited an exothermic binding enthalpy (ΔH) of around −2 kcal/mol, with an intrinsic binding constant (K) of about 2 M^−1^ after correction for electrostatic effects. They also investigated the self-association of the longer Aβ(1–40) in solution and its binding to lipid membranes using calorimetry, observing an approximately linear binding isotherm with an apparent saturation behavior. They proposed peptide penetration into the lipid membrane and peptide aggregation at the membrane surface as possible mechanisms explaining the lipid-induced random coil–β-sheet transition, which is considered an early step in pathogenic plaque formation.

In recent years, Ahyayauch et al. conducted extensive and systematic calorimetric measurements of the interaction between Aβ42 peptide and lipids, particularly in the form of liposomes (large unilamellar vesicles, LUVs) [8,10,11,12]. They found that when LUVs contained sphingomyelin (SM) and cholesterol (Chol) without negatively charged lipids, no measurable heats of interaction with monomeric Aβ were observed. However, reliable measurements were obtained with LUVs containing the negatively charged lipid DMPA, with the peptide association constant (Ka) decreasing regularly with increasing DMPA concentrations. Cardiolipin, another negatively charged phospholipid, favored Aβ42 interaction with bilayers even more than DMPA.

In parallel experiments with L_α_ (liquid-disordered) bilayers, Ahyayauch et al. found that more disordered membranes bound Aβ42 monomers with higher affinity than those in the L_o_ (liquid-ordered) phase. They also explored the influence of gangliosides on Aβ42 interaction with either L_α_ or L_o_ bilayers, finding that GM1 ganglioside facilitated peptide binding to fluid lipid bilayers, particularly at concentrations of up to 3 mol%. Additionally, they studied the effect of the Aβ peptide state of aggregation (monomer, oligomer, and fibril) on its membrane binding capacity. Studies with soluble oligomers have the additional interest that those appear to be most active from the pathogenic point of view [82]. Oligomers and fibrils were able to bind bilayers composed of SM and Chol, with measurable amounts of heat released even in the absence of gangliosides.

From their ensemble of isothermal calorimetry studies, Ahyayauch et al. concluded that Aβ42 fibrils, oligomers, and monomers could spontaneously bind bilayers of various compositions, except that monomers could not interact with SM/Chol binary bilayers. They also found that both enthalpy (ΔH) and entropy (ΔS) were very sensitive to lipid composition, with similar values of Gibbs’ free energy (ΔG) often being attained through compensatory changes in ΔH and ΔS.

### 5.3. Molecular Dynamics (MD) Studies

Yechun Xu et al. were pioneers, in 2005, in using molecular dynamics (MD) to explore Aβ peptides, particularly Aβ40, within explicit bilayer environments [83]. Their research focused on monomeric Aβ40 conformational transitions, revealing a shift from α-helix to coil via helix/β-sheet mixed conformations, suggesting that these intermediates have potential in Aβ oligomerization. They identified four glycines (G25, G29, G33, and G37) that are crucial for β-sheet formation in an aqueous solution. In DPPC bilayers, Aβ40 primarily adopted a helical secondary structure and showed a propensity to reside on the bilayer surface, with Lys-28 being positioned at the bilayer–aqueous interface.

Lemkul and Bevan [62] investigated a similar Aβ40/DPPC system, observing that a segment of the peptide remained embedded in the bilayer, with deeper insertion leading to a near-transmembrane orientation and water molecule association. In shallower insertions, the peptide was strongly associated with the membrane–water interface and phosphatidylcholine headgroups, indicating dynamic Aβ–membrane interactions capable of various conformations. They suggested the need for longer simulation times to observe peptide departure from the bilayer and implied a nucleation site requirement for the aggregation process [62].

Davis and Berkowicz [84] expanded on MD studies, comparing DPPC lamellae with dioleoyl phosphatidylserine (DOPS) bilayers, with the latter featuring unsaturated chains and a net negative charge with Aβ42 peptide. They proposed that Aβ, cleaved from the APP, would interact strongly with the hydrophobic core of zwitterionic bilayers, inhibiting secondary structure changes and promoting aggregation. Conversely, anionic lipid membranes facilitated aggregation by increasing the local peptide concentration and decreasing the surface pH, favoring an Aβ configuration conducive to oligomerization [84]. They did not consider the different states (gel vs. fluid disordered) of DPPC and DOPS bilayers at room temperature in their interpretation.

Ahyayauch et al. [8] delved into the interaction of Aβ42 with mixed bilayers comprising equimolar amounts of SM and Chol, along with varying proportions (5 or 20 mol%) of saturated, negatively charged DMPA. These bilayers were in the liquid-ordered state due to the high concentrations of SM and Chol. MD simulations revealed β-sheet development by the peptide in bilayers containing 5% DMPA, while 20 mol% DMPA maintained a partially helical conformation. This equilibrium behavior aligned with experimental observations indicating maximum β-sheet formation at low DMPA concentrations [8]. Additionally, the study emphasized the significant role of Lys-28 in bilayer binding, which is consistent with prior findings [83].

Gangliosides, known to facilitate Aβ structural conversion and aggregation acceleration, were explored by Manna and Mukhopadhyay [85]. They examined Aβ42 monomers and dimers in GM1 ganglioside-containing liquid-ordered membranes, observing the oligosaccharide head-group of GM1 acting as a scaffold for Aβ-binding through sugar-specific interactions. Aβ dimers exhibited an enhanced β-structure on GM1-containing surfaces, potentially influencing higher-ordered aggregation. These findings were corroborated by Ahyayauch et al.‘s MD studies [10], with POPC bilayers incorporating small amounts of GM1 ganglioside, revealing Aβ42 binding to the bilayer surface without insertion. Limited peptide oligomerization/aggregation was observed under these conditions, supporting the idea of Aβ42 binding facilitated by lipid chain disorder. The dual effect of GM1 on Aβ42 binding, involving ordering properties compensating for the pro-binding effects of a negative charge and H-bonding network, was highlighted [10,85].

**Figure 4:**
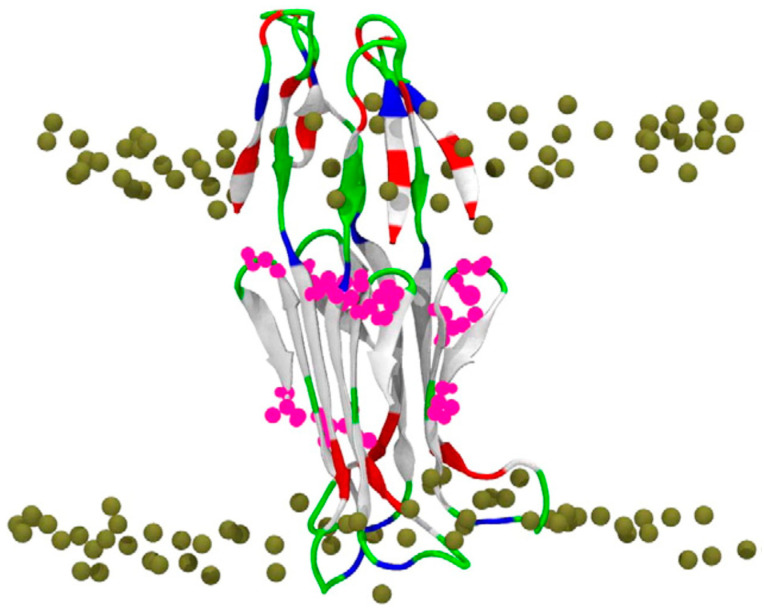
The final state of the 500 ns MD simulation of the Aβ42 β-sheet tetramer in a POPC bilayer. The peptide is shown in cartoon and colored based on the physicochemical properties of the residues: blue, basic; red, acidic; white, hydrophobic; and green, polar. The bilayer phosphorus atoms are shown as Van der Waals spheres in tan color. Lipid tails and water molecules are not shown for clarity. (Reprinted with permission from [86], 2013, Elsevier.)

Poojari et al. [86] employed atomistic MD simulations to explore how Aβ42 behaves within zwitterionic and anionic lipid bilayers. Their simulations included transmembrane β-sheets representing both monomeric and tetrameric forms of Aβ42, as well as a helical structure derived from NMR data. Regardless of the conformation, Aβ42 remained embedded in the bilayer (Figure 4). Interestingly, zwitterionic surfaces and unsaturated lipids were found to enhance the transmembrane stability of Aβ42. The β-sheet tetramer, in particular, exhibited high stability due to inter-peptide interactions. Additionally, the study analyzed the translocation of water within Aβ42-bilayer systems, revealing that the presence of Aβ42 slowed down the water permeation process, with the hydrophobic core serving as the rate-limiting step. The β-sheet tetramer facilitated the passage of more water molecules through the bilayer compared to monomeric Aβ, leading the authors to suggest that membrane-bound Aβ oligomers, rather than monomers, may contribute to the observed permeabilization of membranes.

On the other hand, Brown and Bevan [87] conducted MD simulations to demonstrate the formation of tetramer units by four individual Aβ42 peptides, even in the absence of lipid. These Aβ42 tetramers exhibited a notable increase in β-strand formation relative to monomers, indicating that tetramerization could be a crucial step in fibril formation. Subsequent simulations with bilayers containing pure POPC or a POPC/SM/Chol mixture showed that the tetramer adopted an elongated conformation in the presence of bilayers. Specifically, bilayers containing SM and Chol promoted the formation of a more rod-like structure, which could potentially serve as a seeding point for fibril aggregates.

The presence of tetramers resulted in membranes that were more ordered and rigid, with a greater impact being observed in pure POPC membranes compared to those containing a mixture of POPC, SM, and Chol. In a related study on Aβ oligomerization, Tachi et al. [88] found that Aβ40 initially formed an α-helix, followed by a β-hairpin structure at the air–water interface containing GM1 gangliosides. This β-hairpin structure facilitated the formation of oligomers with intermolecular β-sheets. The results indicate that the process of helix formation, which marks the initial step in the conformational changes leading to pathological aggregation, is initiated at the GM1-glycan moieties rather than at the lipid–ceramide moieties.

Given the substantial decrease in unsaturated lipid contents in the brains of patients with Alzheimer’s disease, particularly in lipids containing (*ω* − 3) docosahexaenoic fatty acid chains [89,90], Hossain et al. [53] demonstrated through spectroscopic and microscopic methods that docosahexaenoic acid inhibits Aβ42 fibril formation. This suggests that membranes rich in polyunsaturated lipids may hinder the formation of Aβ aggregates on their surfaces. Additionally, experiments utilizing electronic spin resonance [75] showed that docosahexaenoic-containing lipids enhanced the interaction of Aβ25−35 peptide with lipid membranes, favoring deep peptide internalization and inhibiting peptide release and subsequent fibrillization.

Furthermore, Ntarakas et al. [91] conducted MD simulations of Aβ1–28 and Aβ26–40 peptides in various lipid bilayers mimicking neuronal membranes in healthy brains and brains affected by Alzheimer’s. These simulations revealed that the presence of polyunsaturated lipids led to stronger adsorption of Aβ peptides to the membrane and weaker binding between peptides when they formed aggregates. These findings support previous experimental results and provide insights into how the lipid composition can influence the behavior of Aβ peptides in neuronal membranes.

In a more recent study by Matthes and de Groot [92], the focus was on understanding the role of Aβ42 oligomer conformations in membrane permeabilization. They discovered that pore formation and ion permeation occurred in the presence of β-sandwich structures with exposed side-by-side β-strand pairs formed by residues 9 to 21 of Aβ42. Importantly, the extent of pore formation and ion permeation was found to depend on the insertion depth of hydrophilic residues 13 to 16 (HHQK domain). These findings suggest that membrane-inserted, layered β-sheet edges serve as a crucial structural motif in aggregate-induced membrane permeabilization.

## 6. An Effort in Understanding

This closing section summarizes a number of data and hypotheses that should be taken in mind for a proper understanding of Aβ peptide binding to cell membranes. General considerations will be discussed with more specific examples provided.

### 6.1. The Validity of Models

Virtually all of the data in this review have been obtained either from model membranes (monolayers, vesicles, and supported bilayers) or from simulation studies. The use of model membranes hardly needs to be justified, so our knowledge on cell membranes that were born in the form of a liposome or in other model studies is vast. Model membranes should not be identified with cell membranes since, as their own name implies, they are intended to be simplified models of the biological structures. Yet simplification is at the core of any science, and cell biology is not an exception.

Computer simulations can be seen as an extreme form of simplification, but here, the following aphorism, attributed to A. Einstein, comes to mind: “Everything should be made as simple as possible, but not simpler”. Lipid bilayer simulations have been in use for the past four decades, and their explanatory and predictive powers have increased in parallel with the availability of more powerful computers. MD, which, in fact, predates computers, has been used to analyze the physical movements of atoms and molecules in membranes. Atoms and molecules are allowed to interact for a fixed period of time, giving a view of the dynamic “evolution” of the system. The time steps in an MD simulation must be short, typically only a few femtoseconds (10^–15^ s) each. Most of the events of biophysical interest, for example, structural changes in lipid chains, take place on timescales of nanoseconds, microseconds, or longer. As a consequence, a typical simulation requires millions or billions of time steps. Currently, advances in computing allow for simulations in the millisecond time scale to be reached with reasonable computation times [93]. Not infrequently, experimental and computational techniques have been applied in parallel in the study of Aβ–membrane interactions, of which some examples are given above. The good correlation of both kinds of results has improved the acceptability of MD simulations in biophysical studies in general.

### 6.2. Aβ Peptide Generation, Membrane Binding, Adsorption, and Insertion

One of the difficulties in assessing the literature in this field is that Aβ peptide generation, membrane binding, and membrane insertion are often confused, although they are structurally and functionally very different, even if some of them may occur almost simultaneously.

(i) Aβ production occurs at the membrane level as a result of the actions of membrane proteases, which cause the release of a peptide in the extracellular aqueous medium. Aβ arises from the processing of the APP precursor protein, a transmembrane glycoprotein that influences neuron growth. APP is hydrolyzed through the sequential activity of β- and γ-secretases, mainly leading to Aβ40 and Aβ42 peptides, with the latter being more hydrophobic and fibrillogenic [94].

(ii) Extracellularly produced Aβ interacts with the cell membrane lipid bilayer [95]. The newly released peptide is hydrophobic enough to partition into the neighboring cell membrane. This is often called “membrane *binding*”, although the name includes two different processes, if not two stages of the same process, specifically peptide *adsorption* onto the membrane/bilayer and peptide *insertion* into the membrane/bilayer. The term *deposition* is sometimes used, mostly meaning adsorption, but its use is not recommended.

(iii) Adsorption depends chiefly on H-bonding and electrostatic interactions (although hydrophobic bonds should not be excluded at this stage). Adsorption is rather easily reversible by, e.g., changing the ionic strength of the media or submitting the membrane–peptide system to gradient centrifugation. The adsorption phenomenon, in the absence of other events, is rather conveniently observed in the Langmuir balance (Section 4.1 and Section 5.1). Ahayayauch et al. [10], using a combination of MD, isothermal calorimetry, and Langmuir balance measurements, observed that Aβ42 peptide adsorbed but did not insert into ganglioside-containing phospholipid membranes in the liquid-disordered state.

(iv) Insertion is an essentially irreversible process dominated by hydrophobic interactions in which the previously adsorbed peptide penetrates the non-polar matrix to interact with the lipid fatty acyl chains [7]. Insertion can be confidently assessed through density gradient centrifugation assays (Section 4.2). A good example of peptide insertion was presented by Ahyayauch et al. [9] using a combination of Langmuir balance, ultracentrifugation, and thioflavin T fluorescence to show the insertion of Aβ42 peptide monomers into sphingomyelin/cholesterol/ganglioside bilayers.

One key consequence of such interactions is that Aβ conformation changes markedly, with an overall increase in the β-structure and a tendency towards oligomer and fibril formation. The latter changes depend on whether the peptide has been inserted into or merely adsorbed to the membrane. β-sheet formation is usually presumed to occur in adsorbed peptides [94]. However, the post-binding effects on Aβ conformation are beyond the scope of this review.

### 6.3. Ordered and Disordered Bilayers

The plasma membrane is laterally heterogeneous (Section 2). The supporting lipid matrix is mostly organized as a continuous fluid-disordered (or liquid-crystalline) L_α_ phase in which discontinuous domains, either L_α_ or L_o_, co-exist. L_o_, or liquid-ordered domains, are rich in Chol. In liquid-ordered domains, the lateral diffusion of the lipid molecules is fast, as in the case of L_α_, while the C-C rotational frequency of the acyl lipid chains is low, i.e., the lipid molecular order is high. Note that the size of the discontinuous domains may be very small compared to the order of nanometers (nanodomains) [20].

The fluidity and molecular order of a membrane, or membrane domain, largely resulting from their lipid composition, greatly influence Aβ binding and subsequent folding/oligomerization. L_α_ bilayers bind Aβ42 peptides with higher affinity than those in the L_o_ phase, but this does not mean that Aβ42 fibrils, oligomers, and monomers cannot, to a smaller extent, bind bilayers in the L_o_ state (Section 5). A previous study [8] intended to clarify a discussion on the influence of the lipid order on Aβ42–bilayer interactions. In summary, Aβ accumulation was related to the presence of cholesterol [96] and lipid rafts [97]. However, other authors [98,99] have challenged the significance of previous experiments. In our opinion, some previous studies [96,97] were, in fact, measuring the joint result of two different processes, namely APP hydrolysis by β- and γ-secretases, yielding Aβ42, and Aβ deposition and aggregation. β-secretase appears to be located preferentially in L_o_ domains [96,100], and thus, Aβ is probably generated in those domains; however, adsorption/insertion and aggregation may occur in different domains. In several of our previous studies [8,9,11,12], Aβ binding to fluid-ordered bilayers was studied in particular detail because of the association of β-secretase to that sort of domains. Interestingly, Ahyayauch et al. [8] found that the Aβ42 affinity, measured, e.g., by isothermal calorimetry, for L_α_ membranes was higher than that for the L_o_ environment in which it is originally generated. Moreover, particular care should be taken to define the aggregation state of Aβ when initially added to the membrane suspension, and monomers should be used when the initial stages of the process are to be described.

### 6.4. Electrostatic Forces

**Figure 5:**
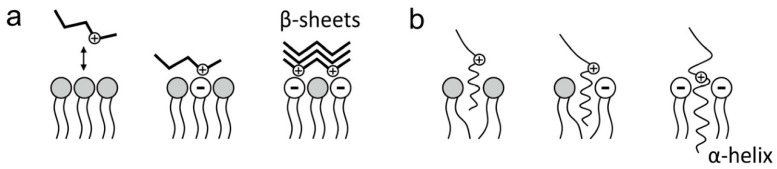
Electrostatic interactions of amyloid peptides with cell membrane lipids. (**a**) Aβ adsorption on the membrane surface; (**b**) Aβ insertion in the membrane. (Taken from [93].)

Phospholipid headgroups are electrostatically charged in general, even if no net electric charge is detected in some of them because of internal salt (*zwitterion*) formation between positive (usually NR_4_^+^) and negative (PO_3_^-^) groups, e.g., PC, PE, and SM. The partial electric charges are enough to direct the binding of some amino acid residues toward the bilayer surface, thus starting adsorption and further putative insertion [49]. Thus, electrostatic interactions influence the attachment of Aβ to the cell membrane. The hydrophilic N-terminus of Aβ contains six positively charged amino acids (including lysine, arginine, and histidine) [101]. The latter may constitute the initial anchor for Aβ peptide adsorption (Figure 5). In particular, MD studies have pointed to Lys-28 as a plausible candidate for the initial anchoring.

Apart from zwitterionic phospholipids, some lipids bearing a net negative charge exist in the plasma membrane that could attract basic amino acid residues in Aβ. Phosphatidic acid and cardiolipin are acidic phospholipids that have been used in experimental studies of Aβ binding [12]. The most abundant acidic phospholipid in the plasma membrane is probably phosphatidyl serine (PS), but it is oriented towards the cytosolic side almost in its entirety, thus having little influence on extracellular Aβ binding. Glycosphingolipids (GSLs), in turn, are phosphate-free lipids, with some of them bearing one or more net negative charges in their sialic acid moieties, as is the case of gangliosides. GSLs exist in the plasma membrane with the large polar headgroup oriented towards the extracellular medium, thus being optimally located for interaction with Aβ. GSLs occur at low concentrations in the membrane, around 5 mol% of all lipids, and not all GSLs bear a net negative charge, but, as seen in many model membrane studies, even a low proportion should be enough to facilitate electrostatic Aβ anchoring.

In relation to the above-mentioned nanodomains (Section 6.3), it should be kept in mind that the heterogeneity of the lipid composition may mean the heterogeneity of the electric charge. It is thus possible that certain domains are enriched in gangliosides, and hence, in net negative charges, which make them good targets for Aβ anchoring. It should be remembered, in this context, the proposed existence of the nanodomains called “lipid rafts”, enriched in cholesterol and GSLs, and are thus in a physical state close to the L_o_ phase [97,102], for which several bilayer compositions used in this kind of studies constitute a good model.

### 6.5. The Role of Specific Lipids

Even if much of this has been already discussed, it is perhaps suitable to review, in the briefest way, the roles of specific lipids in the process of Aβ binding. See Figure 1 for the corresponding structures.

*Phosphatidylcholine (PC).* This is the most abundant phospholipid in mammalian cell membranes, and perhaps the most commonly used one in biophysical studies of Aβ-bilayer binding. The polar group is doubly ionized, forming a *zwitterion*. PC exhibits a marked tendency to self-organize in the form of bilayers (L phases). PC of natural origin (egg and liver) usually gives rise to fluid-disordered L_α_ phases, and the same is true of the synthetic 1-palmitoyl-2-oleoyl and 1,2-dioleoyl derivatives, respectively, POPC and DOPC. Aqueous dispersions of POPC in the form of unilamellar vesicles constitute a simple, satisfactory model for cell membranes. In turn 1,2-dipalmitoyl PC (DPPC) exists in the gel L_β_ phase at room temperature, and it is often employed when, for experimental reasons, this non-physiological condition must be mimicked. Equimolar DPPC/Chol mixtures form good examples of bilayers in the liquid-ordered L_o_ state.

*Sphingomyelin (SM).* This is a structural analog of PC, but a sphingolipid, in which the phosphorylcholine group is esterified to sphingosine C1 (OH) instead of to glycerol C3 (OH). SM is the most abundant sphingolipid in membranes [103]. Its biological activity is rather low, and its main biological role appears to be a sphingolipid depot, from which bioactive lipids, e.g., ceramides or gangliosides, can be readily synthesized in response to a given need. SM of natural origin is often in a gel state or close to the gel–fluid interface at the physiological temperatures of mammals. Equimolar palmitoyl SM/Chol mixtures give rise to robust bilayers in the L_o_ state.

*Gangliosides.* This is a complex group of glycosphingolipids (no phosphorus in their structure) containing one or more sialic acid residues in their glycosyl moieties. In turn, sialic acids, or N-acetylneuraminic acids, are a diverse group of 9-carbon carboxylated monosaccharides, thus endowing gangliosides with one or more net negative charges (Section 6.4). Gangliosides are typically located, albeit at low concentrations, on the outer surfaces of nerve cells, and they are presumed to provide a primary anchor site for Aβ binding. It is possible that Aβ, or at least a portion of it, binds the protruding saccharide portion of gangliosides, which are enriched in domains, just where it is generated, with immediate effects on peptide aggregation; therefore, ganglioside-containing L_o_ domains play a pivotal role in the paradigm of Aβ aggregation.

*Cholesterol (Chol).* This is the only lipid in this short catalog that is not biosynthetically related to fatty acids, and in many senses, it is a most mysterious molecule [104]. Unlike the above lipids, it is a rigid, flat molecule, with its basic structure being constituted by a tetracycle (cyclopentane perhydrophenanthrene). Chol, or close relatives, exist in all eukaryotic membranes. From a biophysical point of view, Chol appears to act as a fluidity buffer, decreasing the fluidity of L_α_ phases and increasing it when added to lipids in L_β_ phases. In fact, when Chol is added to DPPC or palmitoyl SM in the gel phase, a particular fluid phase, namely the L_o_ liquid-ordered phase, is obtained. Even in the absence of other lipids, palmitoyl SM/Chol mixtures can bind Aβ oligomers or fibrils, while pure SM cannot.

### 6.6. Aggregation State

The consensus view of Aβ binding to lipid bilayers is that the process is initiated by an Aβ molecule in monomeric form, probably freshly hydrolyzed from APP at the extracellular side of the plasma membrane. After re-joining the membrane, the peptide (either adsorbed or inserted) will oligomerize and eventually give rise to a fibril and to a plaque. In what is probably a sound strategy, most biophysical studies are focused on the initial stages of the process, and consequently, the vast majority of available data refer to monomeric Aβ interactions with lipids (note, however, that the experimental results reported as being obtained with “monomers” cannot be always guaranteed to arise from oligomer-free samples due to the intrinsic experimental difficulty of obtaining “pure monomer” preparations). Monomer studies should not mask the smaller but significant number of investigations in which pre-formed oligomers or fibrils have been added to lipid bilayers. Note that oligomers appear to be particularly neurotoxic [82,91,105]. Sparing the details of these sometimes complex data, it should be stressed at this point that oligomers and fibrils form and remain in a membrane-bound structure when interacting with bilayers [87,88,105]. According to equilibrium studies (calorimetry and Langmuir balance), binding appears to be entropically driven in most cases due to the peptide disordering the lipid phase upon binding [11]. A naïve interpretation of this observation would be that if Aβ aggregates could not become/remain associated with the membranes, the system at equilibrium would be displaced towards oligomers/fibrils being expelled from the bilayers, a situation that is far different from what we know to be the case. In cell membranes of HEK293 of neuronal origin, oligomeric Aβ42, but not monomers or fibers, has been shown to form voltage-independent, non-selective ion channels, which is suggestive of peptide insertion into the cell membrane [106]. In contrast, Aβ40 oligomers, fibers, or monomers failed to form channels. Diociaiuti et al. [107] proposed that amyloid *prefibrillar oligomers* may constitute a “common structure” of the toxic aggregate, and that the latter may, in turn, elicit a “common mechanism”, leading to neuronal damage in various amyloid-associated neurodegenerative diseases.

### 6.7. Data Integration

Combining the different available data into a single table or figure is a simple but effective way to obtain reliable conclusions. As an example, ultracentrifugation, calorimetric, and Langmuir balance data on Aβ42 in monomer or fibril form, interacting with SM/Chol monolayers/bilayers, composed of SM/Chol ± gangliosides (12 independent parameters, from various publications), were collected and are presented in Table 1. The table could have been made much more extensive, but it is useful in its present form as a methodological example. Several conclusions, which were already discussed above, are seen more clearly here, e.g., (*i*) gangliosides facilitate Aβ42–lipid interaction, (*ii*) fibrils interact with lipids less favorably than monomers, (*iii*) similar association constants, K_a_, are often the result of very different, mutually compensating enthalpic (ΔH) and entropic (ΔS) components, (*iv*) monomers, but not fibrils, become inserted in lipid monolayers at surface pressures compatible with the values found in cell membranes, and (*v*) the fact that Aβ42 monomer interaction with SM/Chol bilayers did not elicit measurable heat exchanges, while ultracentrifugation or Langmuir balance demonstrated a clear interaction, deserves a more detailed investigation, but it may be related to the fact that these bilayers are in the liquid-ordered state. Aβ42 monomer adsorption onto fluid bilayers gives off measurable amounts of heat [10].

## Figures and Tables

**Table 1:** Comparative biophysical data for selective Aβ preparations. Data taken from [9,11,12]. Samples labeled “gang” contain 5 mol% porcine brain total gangliosides. Aβ preparations were either in form of monomers (mon) or fibrils (fib). Average values S.E. (*n* = 3).

Samples				
	SM/Chol (mon)	SM/Chol (fib)	SM/Chol/gang (mon)	SM/Chol/gang (fib)
**technique** **(parameter)**				
Centrifugation (% bound peptide)	41 ± 3.3	-	84 ± 3.9	-
ITC (K_a_, M^−1^, ×10^4^)	-	21.0 ± 0.4	28.0 ± 2	13.0 ± 0.3
ITC (ΔH, kcal/mol)	-	−0.87 ± 0.05	−108.2 ± 12	−29.6 ± 0.4
ITC (ΔS, kcal/mol K)	-	21.6 ± 0.2	−324 ± 8	−71.9 ± 1.8
Langmuir balance (max π_i_, mN/m)	32 ± 0.5	21 ± 0.4	34 ± 0.0	24 ± 0.4

## Data Availability

Not applicable.

## Abbreviations

Chol: cholesterol; CL, cardiolipin; GSL, glycosphingolipid(s); ITC, isothermal calorimetry; PA, phosphatidic acid; SM, sphingomyelin.
